# P-43. Modeling Sepsis Onset Across the Continuum of Care: Prediction in Emergency Department and Hospitalized Patients

**DOI:** 10.1093/ofid/ofaf695.272

**Published:** 2026-01-11

**Authors:** Marco Aurélio Angelo, Bráulio R G M Couto, Débora Vasconcelos, Edna M M Leite, Flávia E B M Pinto, Simony Gonçalves, Walisson Ferreira Carvalho, Naísses Zóia Lima, Rossana Rossana, Samara Mariana M F Silva, Ana Paula Ladeira

**Affiliations:** Hospital Risoleta Tolentino Neves - HRTN, Belo Horizonte, Minas Gerais, Brazil; AMECI – Associação Mineira de Epidemiologia e Controle de Infecções, Belo Horizonte, Minas Gerais, Brazil; Hospital Risoleta Tolentino Neves - HRTN, Belo Horizonte, Minas Gerais, Brazil; Hospital Risoleta Tolentino Neves, Belo Horizonte, Minas Gerais, Brazil; Hospital Risoleta Tolentino Neves - HRTN, Belo Horizonte, Minas Gerais, Brazil; Hospital Risoleta Tolentino Neves - HRTN, Belo Horizonte, Minas Gerais, Brazil; PUC MInas, Belo Horizonte, Minas Gerais, Brazil; PUC MInas, Belo Horizonte, Minas Gerais, Brazil; Biobyte Sistemas Ltda, Belo Horizonte, Minas Gerais, Brazil; Hospital Risoleta Tolentino Neves - HRTN, Belo Horizonte, Minas Gerais, Brazil; Biobyte Tecnologia em Epidemiologia, Belo Horizonte, Minas Gerais, Brazil

## Abstract

**Background:**

Recognizing sepsis as a critical driver of unexpected mortality in both emergency department and inpatient settings, our study seeks to construct a robust model capable of automatically predicting sepsis throughout the continuum of care.

**Table 1:**
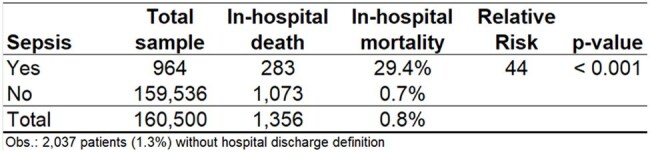
Impact of Sepsis on Mortality: Sepsis patients exhibited a significantly higher risk of death compared to non-sepsis patients.

Sepsis had a devastating impact: 29.4% of sepsis patients died, representing a 44-fold higher relative risk compared with those without sepsis

**Figure 1:**
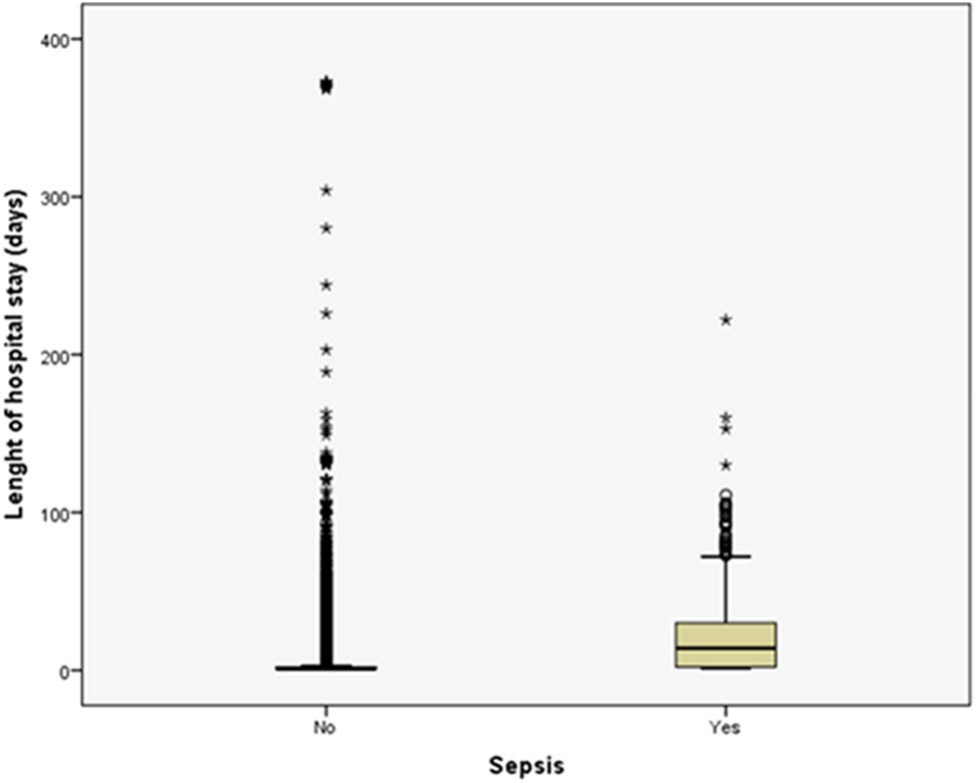
Box-Plot showing the impact of sepsis on the length of hospital stays.

Despite the enormous variability, the length of hospital stay was significantly higher in sepsis cases, with a median of 14 days, compared with a median of one day for non-sepsis patients

**Methods:**

Single-center retrospective cohort study (Jan 2024-Mar 2025) of all emergency department or admitted patients at a public hospital in Belo Horizonte, Brazil (∼3M pop.). Sepsis: positive blood culture OR physician-documented clinical diagnosis (ICD-10 A40/A41). Multivariate logistic regression determined independent sepsis predictors, including 59 exam/assay requests (present/absent during care). The model built was made available as an application within the Datamart A.R.G.U.S.—Assistant for Recovery and Guarding of Urgent and Epidemiological Sentinels—a cloud-based platform hosted on AWS and developed by our team that integrates patient Electronic Medical Records.

**Table 2:**
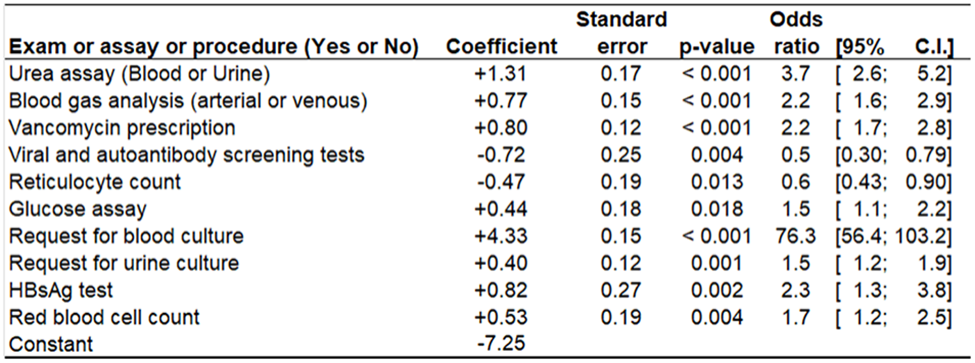
Independent predictors of sepsis identified by multivariate logistic regression among requested exams, assays, or procedures.

The stepwise logistic regression model identified 10 exams, assays, or procedures that, when requested for a patient, demonstrate high predictive ability for sepsis.

**Figure 2:**
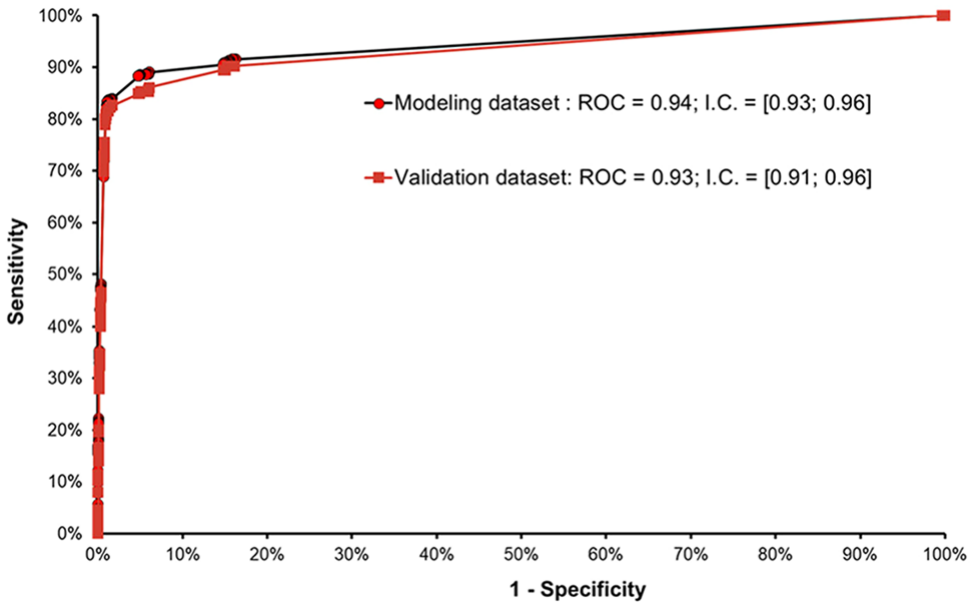
ROC (Receiver Operating Characteristic) curves evaluating the predictive performance of the logistic regression model built to predict sepsis.

The stepwise logistic regression identified 10 readily available exams, assays, or procedures that exhibit high predictive ability for sepsis when requested during a patient's continuum of care

**Results:**

A total of 162,537 patients evaluated in the emergency department or admitted to the hospital: 974 sepsis cases (0.6% risk); 250 cases (26% of sepsis cases) were identified as clinically diagnosed sepsis based solely on ICD-10 codes; 38 cases (4% of sepsis cases) received an ICD-10 sepsis code and had a positive blood culture; and 686 cases (70% of sepsis cases) had a positive blood culture. Sepsis had a devastating impact: 29.4% of sepsis patients died, representing a 44-fold higher relative risk compared with those without sepsis (Table 1). Despite the enormous variability (Fig. 1), the length of hospital stay was significantly higher in sepsis cases, with a median of 14 days, compared with a median of one day for non-sepsis patients. Patients were randomly split into two subsets: 113,424 patients for modeling and 49,113 for validating the logistic model. The stepwise logistic regression model identified 10 exams, assays, or procedures (Table 2) that, when requested for a patient, demonstrate high predictive ability for sepsis (Fig. 2).

**Conclusion:**

Low 0.6% sepsis incidence in a Brazilian hospital contrasted with catastrophic 29.4% mortality (44x higher RR) and prolonged LOS (14 vs 1 day). Logistic regression identified 10 predictive exams/assays, part of a larger effort to optimize the Hospital Infection Control Service's sepsis protocol.

**Disclosures:**

All Authors: No reported disclosures

